# Spatiotemporal monitoring of hard tissue development reveals unknown features of tooth and bone development

**DOI:** 10.1126/sciadv.adi0482

**Published:** 2023-08-02

**Authors:** Marcos Gonzalez Lopez, Barbora Huteckova, Josef Lavicky, Nikodem Zezula, Vladislav Rakultsev, Vendula Fridrichova, Haneen Tuaima, Cita Nottmeier, Julian Petersen, Michaela Kavkova, Tomas Zikmund, Jozef Kaiser, Rupali Lav, Haza Star, Vítězslav Bryja, Petr Henyš, Miroslav Vořechovský, Abigail S. Tucker, Jakub Harnos, Marcela Buchtova, Jan Krivanek

**Affiliations:** ^1^ Department of Histology and Embryology, Faculty of Medicine, Masaryk University, Brno, Czech Republic.; ^2^ Department of Experimental Biology, Faculty of Science, Masaryk University, Brno, Czech Republic.; ^3^ Institute of Animal Physiology and Genetics, Czech Academy of Sciences, Brno, Czech Republic.; ^4^ Department of Orthodontics, University of Leipzig Medical Center, Leipzig, Germany.; ^5^ Central European Institute of Technology, Brno University of Technology, Brno, Czech Republic.; ^6^ Centre for Craniofacial and Regenerative Biology, King’s College London, London, UK.; ^7^ Institute of New Technologies and Applied Informatics, Faculty of Mechatronics, Informatics and Interdisciplinary Studies, Technical University of Liberec, Liberec, Czech Republic.; ^8^ Institute of Structural Mechanics, Faculty of Civil Engineering, Brno University of Technology, Czech Republic.; ^9^ Institute of Histology and Embryology, First Faculty of Medicine, Charles University, Prague, Czech Republic.

## Abstract

Mineralized tissues, such as bones or teeth, are essential structures of all vertebrates. They enable rapid movement, protection, and food processing, in addition to providing physiological functions. Although the development, regeneration, and pathogenesis of teeth and bones have been intensely studied, there is currently no tool to accurately follow the dynamics of growth and healing of these vital tissues in space and time. Here, we present the BEE-ST (Bones and tEEth Spatio-Temporal growth monitoring) approach, which allows precise quantification of development, regeneration, remodeling, and healing in any type of calcified tissue across different species. Using mouse teeth as model the turnover rate of continuously growing incisors was quantified, and role of hard/soft diet on molar root growth was shown. Furthermore, the dynamics of bones and teeth growth in lizards, frogs, birds, and zebrafish was uncovered. This approach represents an effective, highly reproducible, and versatile tool that opens up diverse possibilities in developmental biology, bone and tooth healing, tissue engineering, and disease modeling.

## Teaser

BEE-ST (Bones and tEEth Spatio-Temporal growth monitoring) measures and tracks hard tissue development and healing across species.

## INTRODUCTION

Most of hard tissues in vertebrate bodies are centered on the generation of calcium-based crystalline compounds. This calcium-based biomineralization provides hard tissues, such as bones and teeth, with essential mechanical properties. Therefore, these tissues are responsible for providing a strong supporting body structure, which allows for efficient movement, protects the body surface and internal organs, or, in the case of teeth, ensures hard food processing or predation (*1*, *2*).

Having precise information about growth, regeneration, and biomineralization dynamics in teeth and bones, is one of the basic requirements for research in many fields including developmental biology, tissue healing, bone remodeling, tissue engineering, and disease modeling. One of the major obstacles in current studies is the absence of methodological approaches that enable observation of these dynamic processes, which require high-resolution three-dimensional imaging and temporal information. Current techniques such as micro–computed tomography (microCT), magnetic resonance, or ground sectioning provide only limited end-point information, missing the spatiotemporal dynamics occurring during the growth, regeneration, and remodeling of calcified structures (*3*–*6*).Thus, these approaches do not provide the information uncovering the hard tissue dynamics.

**Fig. 1.**
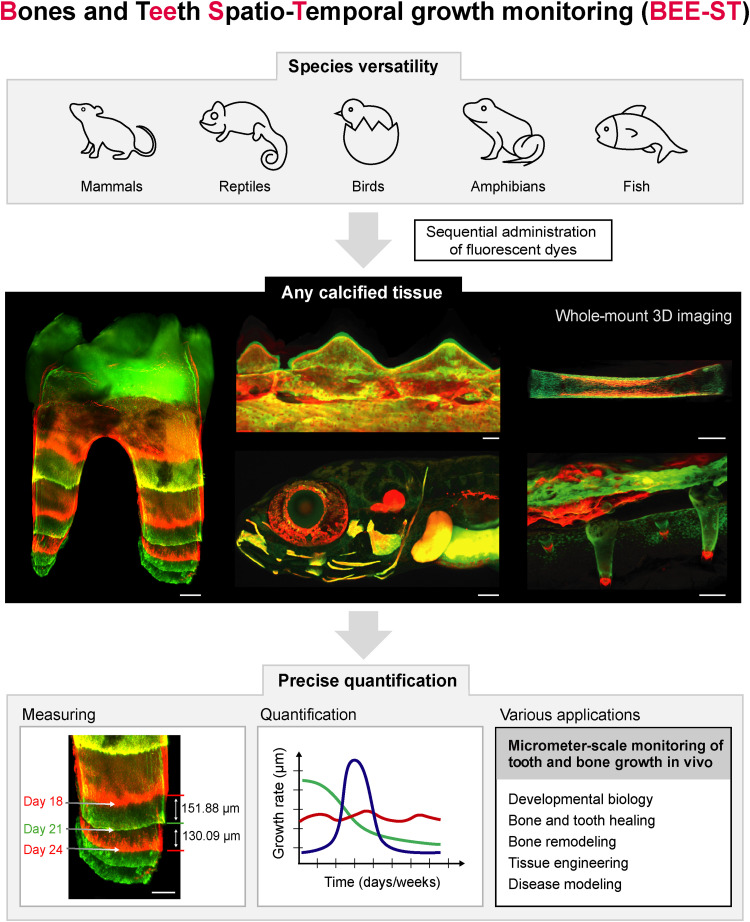
Possibilities of the BEE-ST growth monitoring technique application. We present the BEE-ST approach for tracking the dynamics of hard tissue growth in space and time. Our approach is applicable to all main groups of vertebrates represented by their model organisms: mammals (*M. musculus*), reptiles (*C. calyptratus*), birds (*G. gallus*), amphibians (*X. laevis*), and fish (*D. rerio*). It is based on the sequential administration of calcium-incorporating fluorescent dyes, followed by optical tissue clearing of fully calcified hard tissues, whole-mount imaging, and subsequent precise quantification of the growth in a micrometer scale. BEE-ST has many potential applications in fundamental biology, tissue engineering, or disease modeling. Scale bars, 100 μm. 3D, three-dimensional.

**Fig. 2.**
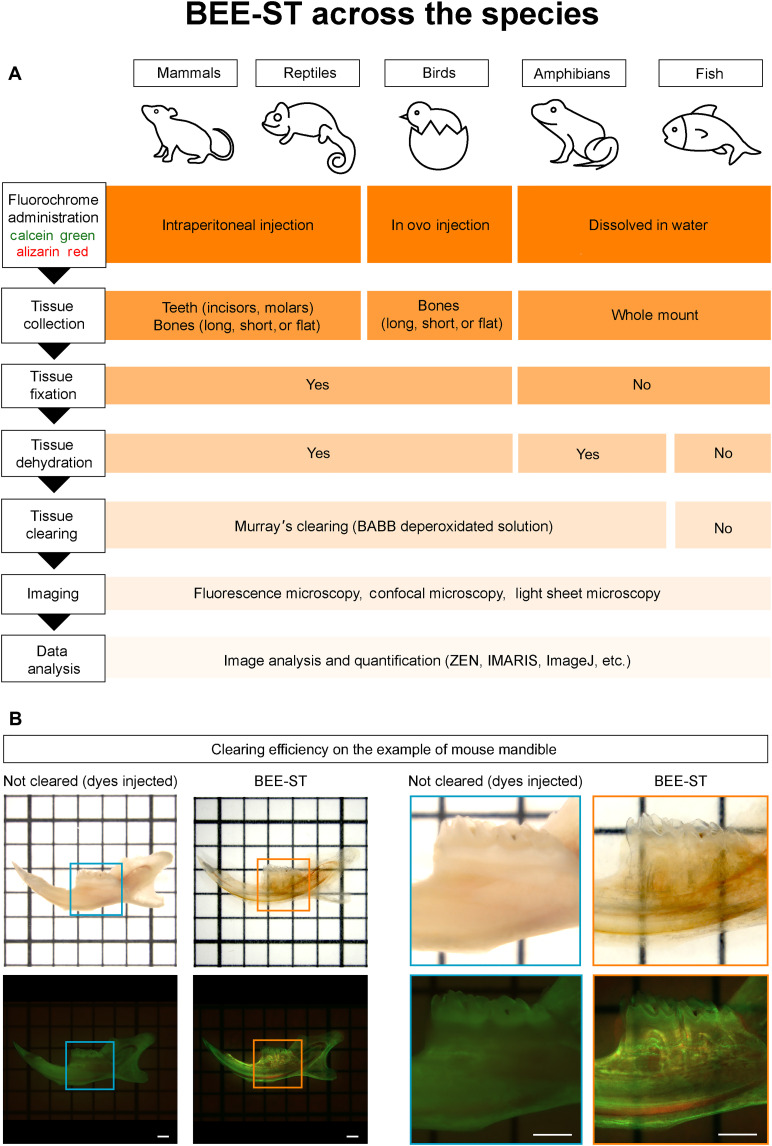
The step-by-step BEE-ST protocol across the species. A detailed protocol summarizing the key steps of the BEE-ST approach in the representative animal models for vertebrates is presented in (**A**). The most effective administration of the dyes is optimized for each species (mammals and reptiles, intraperitoneally; birds, in ovo; amphibians and fish, dissolved in water). For nonaquatic species, it is required to fix and optically clear the dissected calcified tissue, while for aquatic species, this should be omitted. The different samples can be visualized according to the specific needs: wide-field fluorescence microscopy for general scans of larger samples, confocal microscopy for smaller samples and high-resolution three-dimensional imaging, or light sheet microscopy for three-dimensional imaging of larger samples. In (**B**), the efficiency of clearing after using BEE-ST in a mouse mandible taken as an example is shown. The efficiency of BEE-ST staining and clearing is visualized using transmitted light and fluorescent microscope (blue box, not cleared mandible; orange box, cleared mandible). Scale bars, 1 mm.

Here, we have developed an approach that enables monitoring of the dynamics of development, growth, healing, and remodeling of any calcified hard tissues in a three-dimensional context and over long periods of time (Fig. 1) This technique was adapted for selected model species across the vertebrates (*Mus musculus* representing mammals, *Chamaeleo calyptratus* representing reptiles, *Gallus gallus* representing birds, *Danio rerio* representing fish, and *Xenopus laevis* representing amphibians) (Fig. 2A).

Our approach is based on the sequential controlled administration of dyes with the natural ability to incorporate into any currently calcifying tissues at designated time points, followed by the clearing of the nondecalcified hard tissue and high-resolution three-dimensional imaging. The ability of substances to incorporate into forming calcified tissues has been known for more than 50 years, first observed in tetracycline antibiotics (*7*, *8*). These substances not only incorporate into newly forming bone tissues but also incorporate into dentin, enamel, and cementum, if administered during their formation (*9*, *10*). Their fluorescence enables the tracking of the exact site of matrix deposition during the period of their presence after in vivo administration. Dozens of these compounds with different binding efficiencies and fluorescence parameters have been described (*10*). The most commonly used include calcein (green), alizarin (red), xylenol orange, calcein blue, or oxytetracycline (*11*). In contrast to tetracycline antibiotics, these substances have been reported to be harmless in different animal species, such as mice, guinea pigs, amphibians, rabbits, or dogs at effective dosages (*12*–*16*). Their sequential administration enables observation of the dynamics of hard tissue production in a very precise manner by generating stripes, or regions, of fluorescently labeled calcified matrix corresponding to the matrix produced in the short time window after the respective dye was administered. The rapid development of tissue optical clearing techniques in recent years has enabled whole-mount imaging of complex structures and organs, including hard tissues such as bones and teeth (*17*–*21*). In most of these protocols, decalcification step is necessary before the optical clearing of hard tissues. Unfortunately, the decalcification process also removes incorporated dyes from the tissue and, thus, makes it incompatible with the use of aforementioned dyes. However, in addition, there were developed methods that enable the optical clearing of fully calcified tissues allowing the parallel application of dyes incorporating into calcifying hard tissues with three-dimensional whole-mount imaging (*22*, *23*).

Here, we developed a versatile technique named BEE-ST (Bones and tEEth Spatio-Temporal growth monitoring) approach, which takes advantage of whole-mount hard tissue optical clearing that does not require prior decalcification steps to uncover and quantify the dynamics of bone and tooth growth or repair. To image the calcium-binding dyes incorporated at designated time points (Fig. 2B), we adapted modified Murray’s clearing method. This method is based on the usage of deperoxidized mixture of benzyl alcohol and benzyl benzoate (peroxide-free BABB) capable of optically clearing even fully calcified tissue (*22*, *24*, *25*). This was further followed by a nontoxic and device-friendly ethyl-cinnamate for safe imaging. The combination of the fluorescent dyes and calcified tissue clearing techniques enables the precise measuring of the distances between the individual stripes of fluorescently labeled mineralized tissue with reference to the precise time of their injection. Together, this allows for precise quantification of bone and tooth growth and healing in three dimensions on a micrometer scale.

The great advantage of the BEE-ST approach is the nontoxicity at standard concentrations and the versatility of the selected dyes. Moreover, we show that even repetitive administration of selected dyes does not influence calcium deposition or hard tissue morphology. Another advantage is the possibility to obtain three-dimensional information about the hard tissue growth over time without the need for physical destruction of the sample by sectioning or grinding. This method allows whole-mount imaging and, thus, prevents the loss of three-dimensional information and the introduction of artifacts resulting from the physical processing of the tissue before imaging.

In summary, here, we introduce a robust, fast, versatile, and reproducible methodology that enables the quantification of the spatiotemporal dynamics of hard tissues in micrometer scale by a nondestructive approach. This approach was successfully adapted for various model organisms across all major vertebrate phylogenetic groups. Using the example of mouse teeth, unexpected differential regenerative dynamics in continuously growing incisors in young and older animals were uncovered, and the influence of hard and soft food diet on molar root growth was elucidated. We expect that this universal method will find application in fields ranging from developmental biology and tissue repair and regeneration to bone and dental engineering applications.

## RESULTS

### Analysis of the growth dynamics of mouse incisor and molars during aging or hard/soft diet

Continuously growing rodent teeth represent a valuable model to study the development and growth of a complex organ (*26*, *27*). However, analysis of the dynamics of incisor growth throughout an animal’s lifespan, or visualization of sex-dependent growth rate differences, requires more sophisticated methodological approaches.

**Fig. 3.**
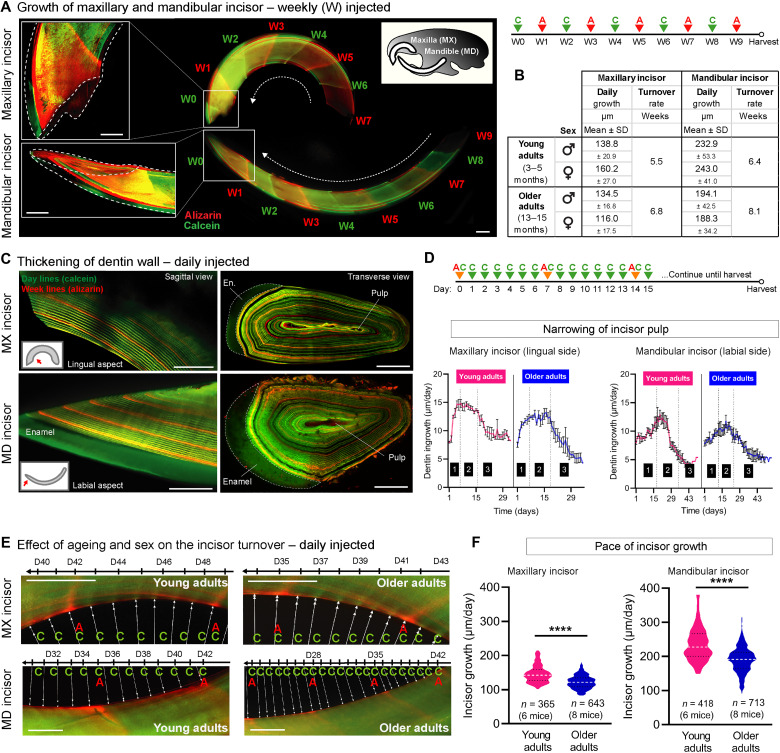
Growth dynamics of the continuously growing mouse incisor. To analyze the growth rate of maxillary (MX) and mandibular (MD) incisors, alizarin (A) and calcein (C) were administered in weekly intervals over 9 weeks (W) (**A**). Quantification of mandibular and maxillary incisors growth revealed that the turnover rates significantly differ between the young adults (3 to 5 months old) and older adults (13 to 15 months old) mice, but no significant alterations were observed when comparing males and females (**B** and **D** to **F**). To analyze the thickening of the dentin wall over time (**C** and **D**) and the daily incisor growth rate (C, **E**, and F), daily calcein administration (with weekly alizarin checkpoints) was performed. Thickening of dentin wall in mandibular and maxillary was demonstrated on the longitudinal and transversal sections (C) and measured from the longitudinal optical sections of both the lingual side (maxillary) and labial side (mandibular) (D). Quantification of the incisor dental pulp narrowing indicates decreased rate of dentin deposition in older mice when compared to young adults (D), *n* = 4 young adult mice (32 stripes); *n* = 4 older adults (45 stripes). This analysis uncovered three stages of dentin wall thickening: (1) acceleration, (2) production, and (3) termination phase that correspond to the distance from dentin-enamel junction and cementum-dentin junction respectively (D). Representative images of daily ingrowths of dentin (incisor growth) shows reduced rate of incisor growth in older animals (E). The reduction of growth rate is more prominent in mandibular incisors (E and F). Data are means ± SD (unpaired Student’s *t* test). ****P < 0.0001. Scale bars, 1 mm (A) and 250 μm (B to H).

**Fig. 4.**
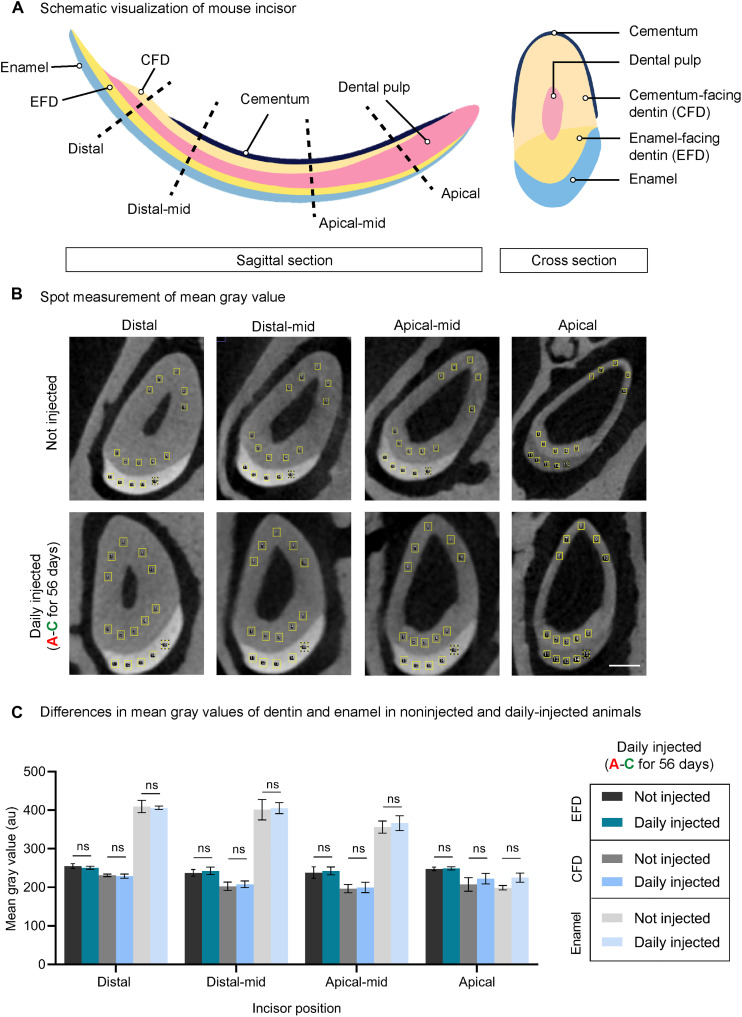
Repeated long-term administration of calcein and alizarin does not affect the mineralization process of hard tissues. Experimental mice were daily alternatively administered by calcein and alizarin for 56 consecutive days (the whole incisor was completely regenerated). Subsequently, the analysis of dentin and enamel density was performed and compared with control mice. (**A**) Longitudinal and transversal sections of a continuously growing mouse incisor and (**B**) the sites of the analysis (distal, distal-middle, apical-middle, and apical regions). The results (**C**) show that daily administration of calcein and alizarin does not cause any significant difference in dentin and enamel density; *n* = 5 per each location, conditions, and the type of analyzed tissue type (in total, 120 measurements). CFD, cementum-facing dentin; EFD, enamel-facing dentin; au, arbitrary units; ns, not significant. Data are means ± SD (unpaired Student’s *t* test). Scale bar, 200 μm.

The BEE-ST approach was used to provide a precise and absolute measurement of incisor growth rate (elongation) and dentin ingrowths (thickening of dentin wall). Calcein and alizarin dyes were alternatingly administered every 168 hours to uncover the dynamics of the renewal rate of the maxillary and mandibular incisors (Fig. 3, A and B). Calcification of dentin occurs in a conical manner, allowing for the growth of the incisors together with the thickening of the dentin wall (Fig. 3C). Daily (precisely every 24 hours) administration of calcein together with weekly alizarin administration (as weekly checkpoints) enabled precise quantification of the incisor growth rate in length, as well as the thickening of the dentin wall using optical cross sections through whole-mount samples (Fig. 3, C to F). Although there were no significant differences observed between the daily growth rates of female and male maxillary incisors (young females: 160.2 ± 27.0 μm versus young males: 138.8 ± 20.9 μm; older females: 116.0 ± 17.5 μm versus older males: 134.5 ± 16.8 μm) or mandibular incisors (young females: 243.0 ± 41.0 μm versus young males: 232.9 ± 53.3 μm; old females: 194.1 ± 42.5 μm versus older males: 188.3 ± 34.2 μm), the growth rate of both sexes decreased significantly during aging. The average mandibular incisor turnover rate was 6.4 weeks in young adults (3 to 5 months) and 8.1 weeks in older adults (13 to 15 months); meanwhile, the maxillary incisor turnover rate in young adults was 5.5 weeks compared to 6.8 weeks required in older mice (Fig. 3B). Analysis of incisor dentin wall thickening after daily calcein administration using optical cross sections uncovered three stages of odontoblast activity throughout incisor development: acceleration, production, and termination (Fig. 3D). Using the mouse incisor, we were able to demonstrate that repetitive use (administered every 24 hours for 56 consecutive days) of selected dyes did not have any adverse effect on the deposition of dentin and enamel (Fig. 4).

**Fig. 5.**
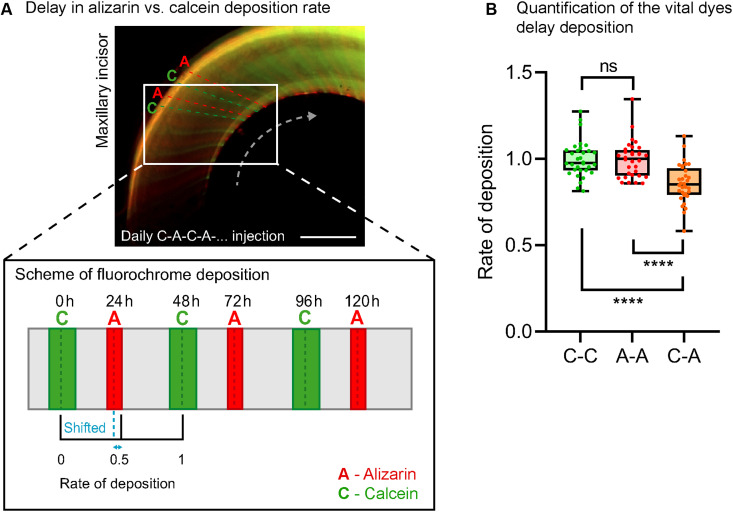
Calcein and alizarin have different kinetics of action in vivo. Alizarin and calcein differ in the duration of deposition in newly formed tissue and the peak of deposition rate (**A**). Calcein provides broader lines than alizarin, indicating its longer duration of activity. (**A**) schematically shows how alternating applications of these dyes create a shift between administrations. This delay was quantified in (**B**) measured in maxillary incisors of daily administered mice; *n* = 32 data points. Data are means ± SD (unpaired Student’s *t* test). ****P < 0.0001. Scale bar, 1 mm.

It was uncovered that alizarin and calcein have different windows of activity after in vivo administration (Fig. 5). Therefore, to quantify daily growth, we used the administration and measurement of only calcein. There was a significant shift (a ratio difference of 0.876, i.e., 2.98 hours in 1 day between the injections) of lines observed in the incisor after the same interval (24 hours) of administration. This difference in incorporation is especially relevant for applications using a daily injection approach and is less significant for longer periods, where distinguishing between different time points by red and green colors brings more benefits.

**Fig. 6.**
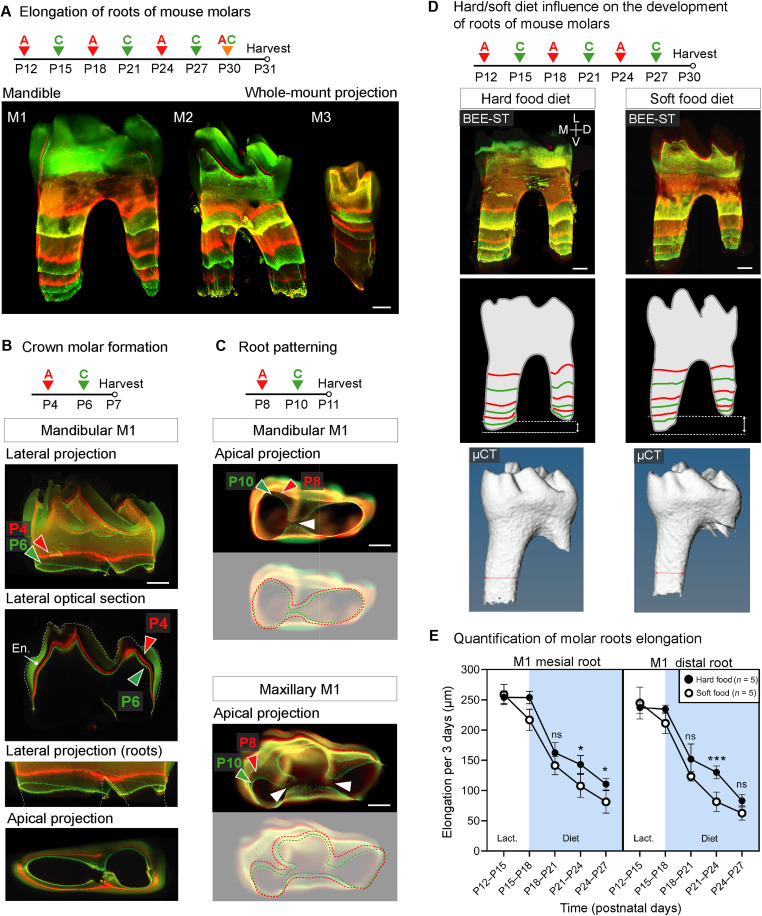
Tracing the dynamics of mouse molar development in time and under the influence of hard and soft diet. (**A**) The elongation of roots of mandibular molars M1, M2, and M3 during an early postnatal (P12 to P30) development visualized by alternating administration of alizarin and calcein in 72-hour intervals. To follow the molar crown (**B**) and root (**C**) patterning, alizarin and calcein were administered in 48-hour intervals at P4 to P6 and P8 to P10, respectively. Whole-mount confocal imaging of cleared sample demonstrates the dynamics of the crown mineralization (B) and the fusion of dentin during root pattering of mandibular (two roots) and maxillary (three roots) first molars (C). (**D** and **E**) The influence of the hard and soft food diet on root elongation during the main period of mandibular first molar growth (P12 to P27); *n* = 5 M1 mandibular molars from hard food mice; *n* = 5 M1 mandibular molars from soft food mice. A hard diet induces faster molar root growth than a soft diet, which causes a slower growth of the roots. M, medial; V, ventral; L, lateral; D, dorsal. Data are means ± SD (paired Student’s *t* test). ***P < 0.001; *P < 0.05. Scale bars, 250 μm.

Because of the lack of methodological possibilities, the spatiotemporal dynamics of root and crown development in mouse molars over time have not been precisely characterized yet. To describe the growth dynamics of molars during development, we sequentially administered alizarin and calcein at different 2- and 3-day intervals during their development (Fig. 6). Our results show the progression of molar root growth in M1 (the first molar), M2, and M3 during an interval between postnatal day 12 (P12) to P30 (Fig. 6A). Before root elongation begins, the dental crown develops. To analyze crown development and early root patterning of the mandibular and maxillary M1, we selected 2-day intervals for the administration of alizarin and calcein (Fig. 6, B and C). Our results reveal that dentin fusion and the formation of two (in the case of mandibular M1) and three (in the case of maxillary M1) roots take place after P10 (Fig. 6C). Using the mandibular M1 as an example of the potential of combining BEE-ST with three-dimensional confocal imaging was shown to allow generation of virtual optical sections to demonstrate growth from different points of view throughout the whole sample (movies S1 and S2). These analyses provide detailed information not only about tooth morphology but also about the dynamics of dentin deposition at selected time points. This allows differences in growth dynamics to be measured under various conditions or in animals of diverse genetic backgrounds. To test our approach, we functionally tested the manipulation of growth dynamics by comparing the effect of hard and soft diets on mandibular molar root growth (Fig. 6, D and E). Both mesial and distal roots showed slower elongation under the soft food diet (Fig. 6E). Therefore, the hardness of the food after the lactation period influences the pace of root growth and, thus, directly affects root length.

### Growth and healing dynamics of mouse long and flat bones

**Fig. 7.**
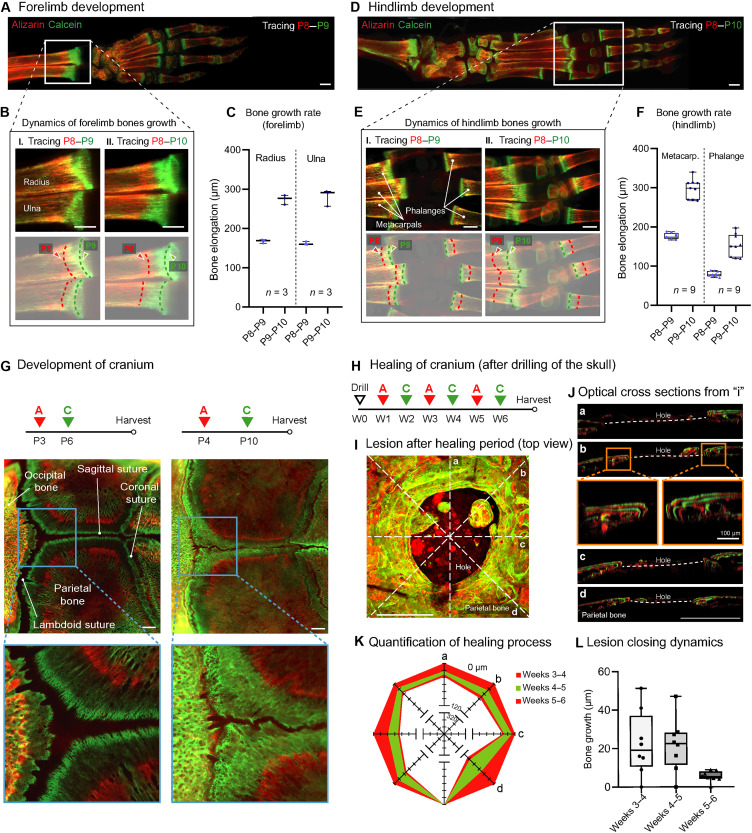
Development and healing of long and flat bones in the mouse. (**A** and **D**) An overview of optically cleared forelimb and hindlimb paws after a series of alizarin and calcein injections during its postnatal development. Quantification of the growth dynamics in forelimb bones (radius and ulna used as example) is shown in (**B**) and (**C**), and that in hindlimb bones (metacarpal bones together with the first phalanges of second, third, and fourth fingers used as an example) is shown in (**E**) and (**F**); *n* = 3 mice traced from P8 to P9; *n* = 3 mice traced from P8 to P10. The BEE-ST approach was also used to trace the development of cranium to observe the dynamics of parietal and occipital bone fusion and the formation of sutures (**G**). The BEE-ST approach was adapted for the quantification of the healing progress after skull injury (**H** to **L**). (I) The partially healed skull 5 weeks after drill (top view). (J) Optical cross sections of the different planes of (I), showing a rounded pattern of bone healing. Quantification (weeks 3 to 5) of the healing progress was performed on the optical cross sections (J) and graphically visualized (K and L). Data are means ± SD. Scale bars, 500 μm.

Uncovering the process of development and healing of long and flat bones is key not only for understanding many developmental abnormalities [e.g., achondroplasia or craniosynostosis (*28*, *29*)] but also for the development of future strategies in tissue engineering (*30*, *31*). First, to enable precise monitoring of the development of long and flat bones in mice, we adapted the BEE-ST approach for use in newborn mice and pups (Fig. 7, A to G, and table S1). Using the example of the bones that build the forelimb and hindlimb, the development of bones in a single animal over time was analyzed, showing that the rate of bone growth is a nonlinear function of time (Fig. 7, C to F). To achieve this, we selected sequential administration of alizarin and calcein during the time points P8 and P10 and measured daily ingrowths in the radius, ulna, metacarpal bones, and phalanges (Fig. 7, A to F). The BEE-ST approach can also be used to monitor the development of flat bones using the example of cranial bone fusions in newborns, when administered in the P3 to P10 period as shown in Fig. 7G. Furthermore, the dynamics of the fusion of the parietal bones with the occipital bone, forming the sagittal and lambdoid sutures, were elucidated using BEE-ST (Fig. 7G). This is particularly important for understanding the dynamics of some congenital malformations where the cranial sutures fuse prematurely, such as in the case of craniosynostosis (*29*). Last, the BEE-ST approach was adapted to enable the monitoring of cranium healing after skull drilling at weekly time points and established a method for the quantification of the lesion healing (Fig. 7, H to L). The method enables quantification of the progress of bone healing over space and time (Fig. 7, K to L). This was achieved by the measurement of weekly growth lines from optical sections taken by three-dimensional confocal imaging (Fig. 7J). The results highlight that the healing of flat bones after injury slows down over time (Fig. 7, K and L). This approach will be instrumental for study of the bone healing process and for the rapidly growing field of bone tissue engineering enabling to evaluate the dynamics and the efficiency of the hard tissue–forming process on scaffolds both in vitro and in vivo.

### Monitoring the development of teeth and bones in reptiles and amphibians

**Fig. 8.**
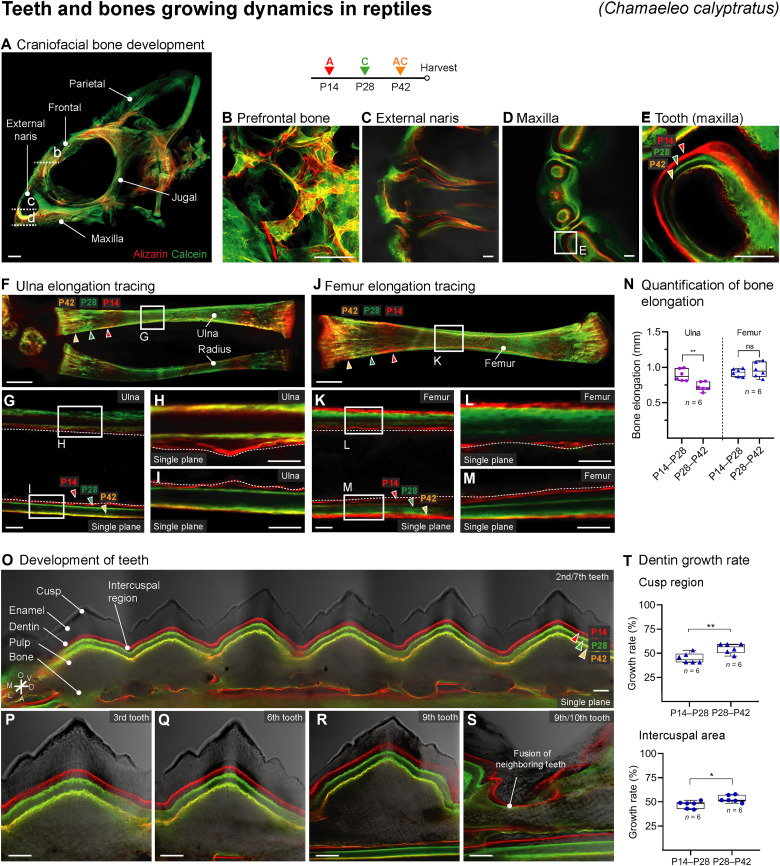
Dynamics of chameleon (*C. calyptratus*) skeletogenesis and odontogenesis. (**A**) The progress bone formation in a whole-mount chameleon with a focus on the craniofacial bones: prefrontal bone (**B**), external naris (**C**), maxilla (**D**), the tooth (**E**); and long bones (**F** to **N**). Quantification of long bone elongation (ulna and femur) is shown in (N); *n* = 3 chameleons (six ulnae and six femurs). The growth rate of dentin was analyzed using 14-day intervals and showed dentin deposition over time across teeth from the mandible (**O** to **T**). The dentin mineralization is coordinated along the jaw (O to S), but the rate of dentin growth showed different deposition patterns in the cusp and intercuspal regions in time (T); *n* = 1 chameleon (six teeth cusps regions and six interdental regions). Data are means ± SD (unpaired Student’s *t* test). **P < 0.01; *P < 0.05. Scale bars, 1 mm (A) and 100 μm (B to S).

**Fig. 9.**
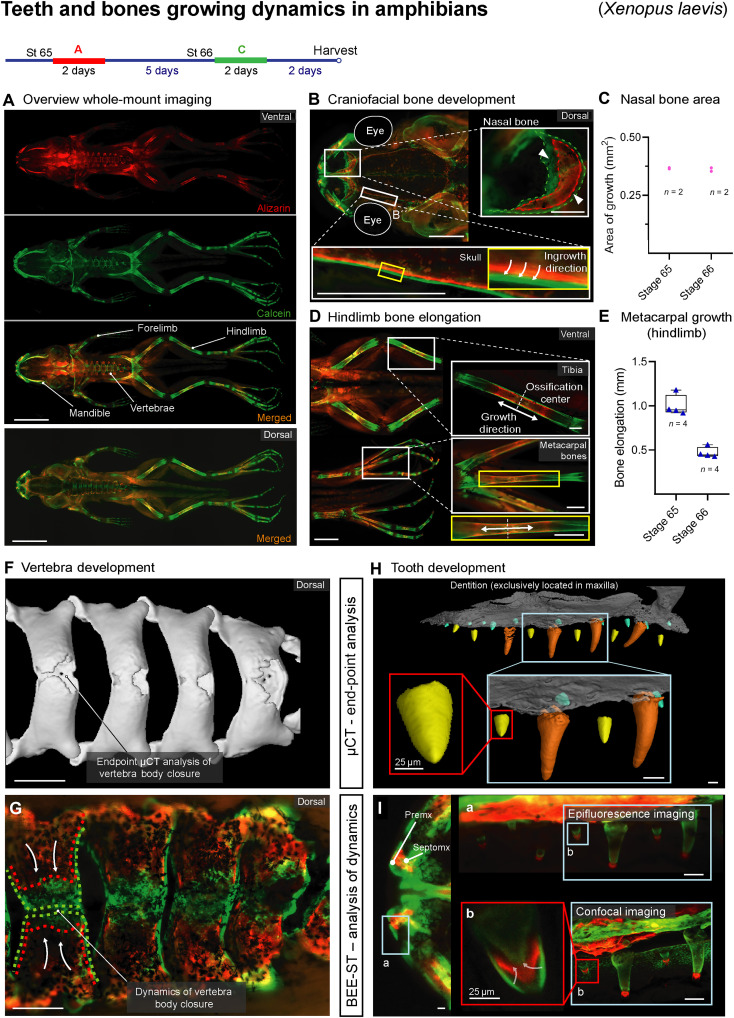
The versatility of BEE-ST shown in amphibians’ (*X. laevis*) bone and tooth growth. (**A**) Whole-mount frog images in separate and merged channels from both ventral and dorsal views after alizarin and calcein administration into the water. The dynamics of the formation of craniofacial bones (**B** and **C**) visualized the growth of the nasal bone in high detail; *n* = 1 frog (two nasal bones). (**D** and **E**) The dynamics of hindlimb bone elongation that enables the detection of the ossification center and the direction of growth of long and short bones shown in the tibia and metacarpal bones; *n* = 1 frog (four metacarpal bones). Comparison of microCT and BEE-ST approaches used for tracing of the development of vertebrae (**F** and **G**) and maxillary dentition (**H** and **I**). Subset (a) represents panoramic fluorescence imaging of the upper jaw and (b) shows a spatiotemporal reconstruction of tooth mineralization (confocal imaging). Data are means ± SD. Scale bars, 5 mm (A), 2 mm (B and D), 500 μm (F and G), 100 μm (H, I, and a), and 25 μm (b).

Compared to *M. musculus* (representing mammals), little is known about the development of bones and teeth in other major vertebrate groups. Here, the versatility of the BEE-ST approach using *C. calyptratus* (representing reptiles) and *X. laevis* (representing amphibians) is demonstrated (Figs. 8 and 9). To observe the development of chameleon bones and teeth, we administered the dyes at 2-week intervals: calcein at P14, alizarin at P28, and both dyes to obtain a pseudo-orange color at P42. Confocal imaging of optically cleared hard tissues then enabled us to observe and measure multiple aspects of the development of various bones (Fig. 8, A to N) and teeth (Fig. 8, O to T). Monitoring of tooth development uncovered a joint mineralization of dentin in several neighboring teeth (Fig. 8S). Quantification of the dentin growth rate in the cusp and interdental region revealed that the dentin deposition rate increased slightly during the traced weeks 4 to 6 compared to the interval from weeks 2 to 4 of postnatal development (Fig. 8T). The three-dimensional analysis uncovered unreported parameters not only of the dynamics of tooth growth but also of the process of ankylotic fusion of the tooth and bone (Fig. 8, O to S, and movie S3).

Amphibians have traditionally been used in developmental biology especially for the study of their specific features of early embryogenesis (*32*, *33*). However, not much is known about the dynamics of amphibian bone and tooth development. The BEE-ST approach was adapted for use with these animals by adding alizarin or calcein to the water tanks for 2 days, alternating with a 5-day period with no dye present in the water during the postmetamorphosis developmental stages 65 to 66 (Fig. 9 and table S1) (*34*). Subsequent whole-body fluorescence imaging showed bone growth at different locations, which revealed the directions of bone growth and the thickening of the bone wall. Furthermore, it enabled us to quantify these processes within each individual animal (Fig. 9, A to F). The bodies of vertebrae were shown to dynamically fuse, which could be compared to data from end-point microCT analysis. This illustrates the possibility of combining the two methods to obtain both the information about the bone growth dynamics via the BEE-ST and precise volumetric data via microCT (Fig. 9, F and G), therefore enabling the generation of comprehensive sets of data from a single sample. Last, we focused on one of the least studied structures of *X. laevis*—the teeth, which are located exclusively in the maxilla (Fig. 9, H and I) (*35*). The analysis uncovered similarities in the dentin growth process in different rows of teeth that differ in size. Together with microCT analysis, the BEE-ST approach provides a comprehensive view of the complex organization and dynamics of development of amphibian teeth (Fig. 9, H and I).

### Bone development and remodeling in ray-finned fish and birds

**Fig. 10.**
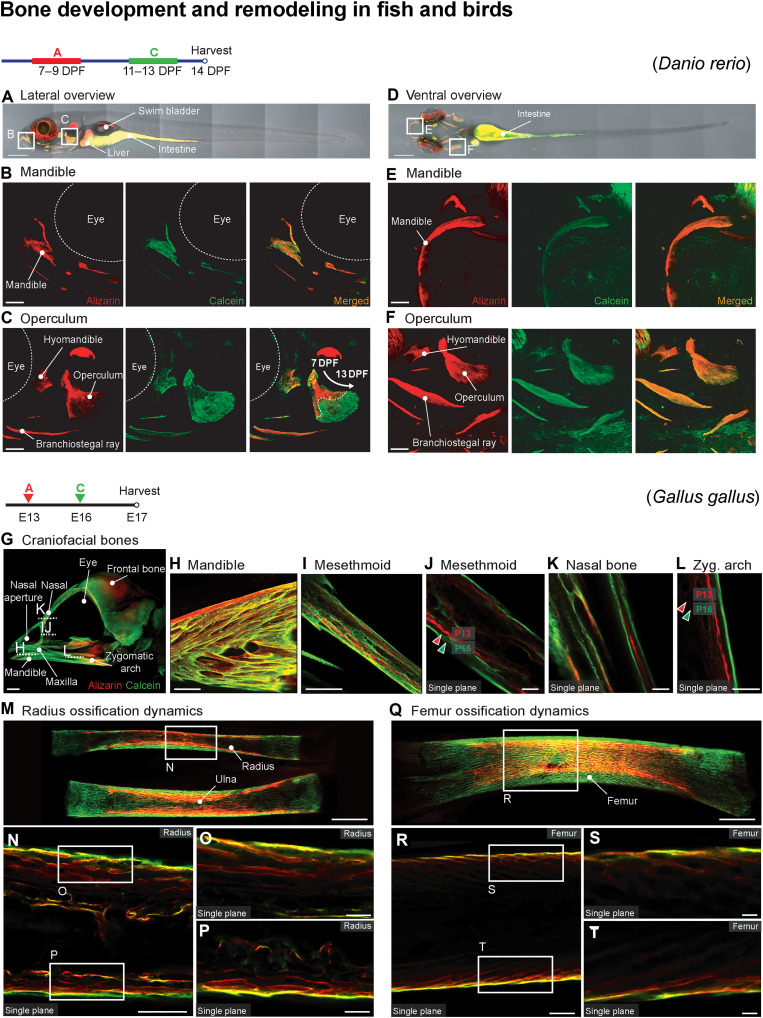
Adaptation of BEE-ST for use in zebrafish (*D. rerio*) and chicken (*G. gallus*). Sequential administration of calcein and alizarin into the water of experimental zebrafish larvae enables us to trace the development of different bones forming the head: lateral whole-mount overview (**A**), lateral view of the mandible (**B**) and operculum (**C**), ventral whole-mount overview (**D**), and ventral detail of the mandible (**E**) and operculum (**F**). In the example of the chicken (*G. gallus*) embryo, we show the possibility of using the BEE-ST approach also to following the development of bones of birds by in ovo administration of calcein and alizarin. (**G**) The development of craniofacial bones (lateral whole-mount skull overview), the mandible (**H**), mesethmoid (**I**), mesethmoid—single plane (**J**), nasal bone—single plane (**K**), zygomatic arch—single plane (**L**), radius ossification dynamics (**M**), details of radius single planes (**N** to **P**), femur ossification dynamics (**Q**), and femur single planes details (**R** to **T**). Scale bars, 250 μm (A, D, H, I, N, and R), 50 μm (B, C, E, F, J, K, L, O, P, S, and T), 2 mm (G), and 100 μm (M and Q).

In the past decade, zebrafish (*D. rerio*) has become an important model organism used for the study of bone development and disease modeling (*36*). To enable the study of dynamics of bone growth and development in this model organism, we further adapted the BEE-ST approach (Fig. 10, A to F). In zebrafish, most bony tissues arise from condensed mesenchymal precursors that differentiate directly into osteoblasts, which generate the bone by intramembranous ossification. Craniofacial bones start to form around 3 to 4 days postfertilization (DPF), and this process continues until adulthood (around 90 DPF) (*36*). As the zebrafish body is already transparent, in contrast to other tested organisms, zebrafish larvae were imaged as living organisms without the need for tissue fixation and optical clearing steps (Fig. 2A). To observe craniofacial bone development, we selected a 48-hour period of exposure to alizarin (7 to 9 DPF). This was followed by a 48-hour period without the presence of a dye, followed by a 48-hour period (11 to 13 DPF) with the presence of calcein in the water. Deeply anesthetized animal was then imaged from lateral and ventral perspectives as a whole-mount in three dimensions using a confocal microscope (Fig. 10, A and D), and the dynamics of growth of bones in the head including the mandible, hyomandible, and operculum were analyzed (Fig. 10, B, C, E, and F). In principle, this approach would also be applicable for monitoring the development of long bones (e.g., ribs), spine, or fin bones, simply by choosing different time points of exposure to the dyes. Anesthetizing the experimental animal without the need of fixation and subsequent optical clearing provides the potential to allow the experimental animals mature after the initial imaging, reanalyze it at later stages, and, thus, determine the fate of the deposited tissues at future time points.

Birds (Aves) represent the last class of vertebrates whose bone development dynamics were investigated. The BEE-ST approach was further adapted to be usable for studying bone development during in ovo embryogenesis. Chicken (*G. gallus*) was used, as it is the most used species in avian developmental biology (Fig. 10, G to T) (*37*). To achieve optimal signal in the experimental embryo, we injected dyes in ovo directly into the abdomen of the embryo. Time points of administration of alizarin and calcein at embryonic day 13 (E13) and E16, respectively, were selected to observe the development of the head and selected long bones such as femur and radius. The embryos were then collected at E17. This enabled to observe the growth of selected bones including the nature of their bone wall thickening (Fig. 10, M to T).

Overall, we have adapted the newly developed BEE-ST approach to all major vertebrate groups that represent the most used model organisms in research. The results demonstrate the versatility of the approach and open diverse avenues for the analysis and precise quantification of bone and tooth growth dynamics during development, remodeling, or repair under physiological or pathological conditions.

## DISCUSSION

Here, we introduce a versatile approach (BEE-ST), which enables the tracing of the processes of in vivo mineralization in space and time. This approach opens a variety of possibilities in multiple fields focused on bone or tooth development, regeneration, and healing. In addition, its use can be extended for bone and tooth tissue engineering.

Current methods used for the imaging of the hard tissues are in the vast majority based on microCT imaging (*3*). This imaging provides a three-dimensional perspective of observed objects with the spatial resolution dependent on the size of the observed object (*38*). Although microCT imaging can provide relatively high-quality spatial resolution, it enables to scan only one time point of the observed animal. To trace the dynamics of the process, more experimental subjects must be analyzed, which brings the individual variability into the measurement. In the case of bone remodeling or continuous tooth growth, a standard microCT analysis does not provide the information needed to uncover the tissue growth dynamics. However, in some specific cases, it has been shown that microCT or high-resolution synchrotron radiation–based microCT analysis can answer some questions about hard tissue dynamics by following their gradual ingrowths. This has been particularly demonstrated, for example, in the animal fossils when focusing on the cementum growth lines that correlate with metabolic rate (*39*).

The BEE-ST approach uncovers the mineralization dynamics at multiple selected time points within a single experimental animal in a single scan. The data are obtained by whole-mount, nondestructive three-dimensional confocal or light sheet imaging providing excellent spatial and temporal resolution. Thus, BEE-ST can be used to uncover the dynamics of the hard tissue growth and remodeling or even to investigate healing patterns after the injury. This is ensured by the sequential administration of harmless fluorescent dyes that incorporate into hard matrices that are being mineralized in that specific moment. This is followed by a nondecalcified tissue clearing and whole-mount three-dimensional imaging that enables the quantification on a micrometer scale.

The use of calcium-binding fluorochromes in hard tissue research has been known for several decades and has been used in various adaptations (*40*, *41*). Traditionally, they were used for the visualization of the calcified tissue after its isolation from the experimental animal or in the cell culture for uncovering signs of mineralization (*42*). Later, in larger mammals (pig and sheep), these dyes were administered in vivo, and the samples were subsequently processed by destructive ground sectioning to observe the development of mineralized tissues in single plane (*43*, *44*). Recently, calcein and xylenol orange administration followed by fluorescent imaging was also used to follow the natural replacement dynamics of gecko teeth (reptiles) (*45*). In bone field, the administration of hard tissue–incorporating fluorochromes has been used to observe bone healing after the injury and to assess the integration of artificial implants using wide-field fluorescence imaging (*46*, *47*). In addition to these studies, BEE-ST represents a universal approach broadly applicable in variety of species providing a tool to precisely measure the dynamics of hard tissue growth and healing in a three-dimensional perspective.

Although, nowadays, a variety of different dyes with the abovementioned features were described, not all of them have optimal properties. To achieve optimal results, the sequential administration of two carefully selected dyes, alizarin red and calcein green, was used. Both are in different fluorescent spectra and provide a strong and highly specific fluorescence signal with low background (*48*). In addition, in some cases, a third pseudo-orange signal can be obtained while using the combination of calcein and alizarin via their parallel administration. This is specifically useful while studying calcified tissues that are not being remodeled over time. Moreover, it was shown that the selected dyes do not disrupt the process of biomineralization even during long-term daily administration. Furthermore, at the selected concentrations, all dyes are nontoxic for the animals (*49*).

An unknown fact is that fluorescent dyes capable of incorporating into currently calcifying tissues have different in vivo kinetics. We revealed that even alizarin and calcein exhibit a slightly shifted pattern in the forming dentin of mouse incisor that grows at stable pace. Via the use of continuously growing incisors, this shift was demonstrated and quantified in detail. This is particularly important to consider when the analysis needs to be performed while using 24 hours or in even shorter intervals for dye administration. When the intervals are 48 hours or more, the differences are negligible, and it might be more beneficial to use a combination of dyes. Thus, it is crucial to carefully select dyes and consider their combinations accordingly for each type of experiment.

Using continuously growing mouse incisor as a model, the BEE-ST approach was used to precisely quantify several features of its growth. We used both weekly and daily administrations of dyes to quantify the speed of incisor growth, turnover, and dentin wall thickening speed in groups of animals with different ages and sexes. This was achieved by daily calcein administrations together with combined alizarin and calcein weekly administrations used as checkpoints. Compared to previous studies, we were able to quantify the growth of the incisor with several orders of magnitude more precise manner in real numbers (*50*). This approach can be used for a precise quantification of incisor growth acceleration after its injury (shortening by the tooth clipping). Previously, it was only possible to observe this by making notches in the enamel and subsequently following the changes in their location as the tooth regrows (*51*). These measurements unfortunately did not provide real values but only a relative comparison to the neighboring tooth. Moreover, notches in teeth cause damage to the enamel and sometimes even dentin that might influence the experiment. In the example of mouse molars (teeth more similar to the human dentition), the BEE-ST approach can be applied to describe and quantify root patterning and elongation during their postnatal development. The speed of root growth was found to depend on the type of diet (soft or hard food) given to the pups during the postlactation phase. These results may have implications for human medicine and may explain the mechanisms controlling root elongation.

We have shown that the BEE-ST approach can be independently used in all major groups of both terrestrial and aquatic vertebrates to study processes such as bone development, formation of ankylosis, or wound healing. We present a complex, efficient, and very precise approach that not only is widely applicable in vivo in various animals but also has potential to be used in tissue engineering and can be combined with other techniques.

## MATERIALS AND METHODS

### Vital dyes preparation

For all animal models, the dyes were prepared as follows: calcein (0.9 mg/ml; C0875, Sigma-Aldrich) and alizarin red S complex (4.5 mg/ml; A5533, Sigma-Aldrich). Solutions were prepared in 2% NaHCO_3_ (71628, Honeywell Fluka) in MilliQ water, filtered using a syringe filter 22 μm polyethersulfone membrane (99722, TPP) under sterile conditions and stored at 4°C in the dark until its use (up to 1 month). The volume and ratios of injections for the different animal models are specified in table S1.

### Hard tissue clearing

BABB peroxide–free clearing solution was prepared by mixing benzyl benzoate (B6630, Sigma-Aldrich) and benzyl alcohol (10880, Penta) in a 2:1 ratio and adding activated neutral Brockmann Grade I 58 Å aluminum oxide (11502.A1, VWR) at a concentration of 0.25 g/ml. The mixture was agitated for at least 1 hour up to overnight at room temperature (RT). Then, the clearing solution was centrifuged at 2000*g* for 10 min. The supernatant was used in the optical clearings. Protocol was adapted from (*25*).

### Mouse (*M. musculus*)

All experiments with mice were approved by the Ministry of Education, Youth and Sports, Czech Republic (MSMT-8360/2019-2, MSMT-9231/2020-2, and MSMT-6379/2022-4). Mice were kept in a 12-hour:12-hour light/dark cycle, 18° to 23°C, and 40 to 60% humidity. Animals had access to food and water ad libitum. No exclusion criteria were applied for experimental animals.

### Hard/soft food–type experiment

A soft diet (Nutra-Gel, Bio-Serv) or hard diet (regular 10 mm pellet food, 1414, Altromin) was given to the newborn mice since P10 in a specialized pyramid feeder located inside of the cage. The dam was removed from the cage at P17. Food was routinely changed every 3 days.

### Tissue preparation and imaging

Mouse molars and incisors were carefully isolated under stereomicroscope and fixed in 4% paraformaldehyde (PFA) (18070, Penta) prepared at pH 7.4 for 2 hours at RT. Bones were fixed in 4% PFA overnight. Fixed tissues were subsequently dehydrated in an ethanol gradient (30 to 50 to 80 to 100%) diluted in MilliQ water (15 min in each concentration for teeth and 1 hour for bones) and stored overnight in 100% ethanol at RT. Excess ethanol was removed, and samples were transferred to the prepared BABB peroxide–free clearing solution. The incisor, molar, or bone samples were incubated for 72 hours at RT and slowly shaken in the prepared BABB solution, until reaching complete transparency. For imaging, samples were transferred to ethyl cinnamate (W243000, Sigma-Aldrich) and imaged in a suitable vessel: glass-bottom dish for a fluorescence microscope or μ-slide ibidi dish (80826 and 80281) for confocal microscope.

### Skull drilling

Six-month-old mice were anesthetized by an intraperitoneal administration of a mixture of ketamine/xylazine (90 and 12.5 mg/kg, respectively) in 0.9% saline. Fur was removed from the head, and the area was swabbed with 70% ethanol. The skin was subsequently cut, and the injury was generated in the middle of the parietal bone using a drill machine without damaging the underlying tissue. Carboxymethylcellulose (Ocugel) drops were topically applied to the eyes of the mice before and after surgery. Ibuprofen (40 mg/kg) was dissolved in the water tank as analgesia for a week after surgery.

### X-ray microtomography

Before the scanning the samples were placed in either a 2 or 15 ml tube (based on sample size) and mounted in 1% agarose gel to prevent the motion of the sample during the scan. The microCT measurement was performed using the GE Phoenix v|tome|x L 240 laboratory system equipped with a 180 kV/15 W nanofocus x-ray tube and a 4000 × 4000 pixel flat panel detector with a pixel size of 100 μm. The scan conditions were as follows for both jaw and spine samples: The samples were scanned at 60 kV acceleration voltage and 200 μA tube current, and the exposure time of 500 ms and three images were averaged to reduce the noise. Over the 360° rotation of the sample, 2700 projections were taken in the case of the sample of the jaw (with a 3.4 μm voxel resolution of the scan), and, in the case of the sample of the spine, 2700 projections were taken, and the final scan resolution was set to the 15 μm voxel size. The tomographic reconstruction was performed by the GE phoenix datos|x 2.0 software. The reconstructed data were imported into the VG Studio MAX 2022.1 software where all the data visualizations were created.

### Chameleon (*C. calyptratus*)

All experiments on chameleon juveniles were approved by the Ministry of Education, Youth and Sports, Czech Republic (MSMT-10946/2021-5) and followed national regulations. Juvenile animals were obtained from private and commercial breeders. All chameleons were kept in a 12-hour: 12-hour light/dark cycle, 25° to 30°C, and 55 to 65% humidity. Animals had access to food and water ad libitum. No exclusion criteria were applied for experimental animals. At selected time points, animals were euthanized, decapitated, and fixed in 4% PFA for at least 48 hours. Jaws, skull, and limbs were isolated. Fixed tissues were processed through a dehydration ethanol gradient (30 to 50 to 80 to 90 to 95 to 100%) at RT (each for 1 hour) and then placed directly into the prepared BABB solution. Teeth and bones were kept separately depending on the size of the sample and achieved complete clearing for 24 to 72 hours.

### Frog (*X. laevis*)

The work with *X. laevis* was carried out according to the Czech animal use and care law and approved by the local authorities and committees (MSMT-30784/2022-1; Animal Care and Housing Approval: 45055/2020-MZE-18134, Ministry of Agriculture of the Czech Republic; and Animal Experiments Approval: CZ 62760214, State Veterinary Administration/Section for South Moravian Region). No exclusion criteria were applied for experimental animals. The generation and cultivation of *Xenopus* embryos were performed following general protocols. Briefly, testes from males under anesthesia (20% MS-222, A5040, Sigma-Aldrich) were removed surgically from the body cavity and transferred to cold 1× Marc’s Modified Ringers (MMR; 100 mM NaCl, 2 mM KCl, 1 mM MgSO_4_, 2 mM CaCl_2_, and 5 mM Hepes, buffered to pH 7.4) supplemented with gentamycin (50 μg/ml; G3632, Sigma-Aldrich). To induce egg laying, fully mature *Xenopus* females were injected with 260 U of human chorionic gonadotropin (Merck, Ovitrelle 250G) into the dorsal lymph sac for about 12 to 16 hours before use and kept overnight at 18°C. For fertilization, eggs were squeezed from an induced female directly into a petri dish and mixed with a piece of testes in 0.1× MMR. Then, embryos were cultivated in 0.1× MMR at 18° to 21°C. Once the embryos reached the desirable stage to trace the calcium deposition, they were transferred to a water tank containing the fluorochrome dyes dissolved in the water. At the selected time point, the frogs were euthanized and sacrificed. Skin, inner soft organs, and blood vessels were carefully removed using scissors and tweezers under stereomicroscope. Frogs were staged after the normal table of Nieuwkoop and Faber (*34*).

### Chicken (*G. gallus*)

All experiments on chicken embryos were performed according to national regulations. The project complies with Act No. 246/1992 Coll. on the Protection of animals against cruelty, as amended. Experiments with chicken embryos were approved by the expert committee for ensuring the welfare of experimental animals at the Breeding facility for laboratory animals of lower vertebrates at the Faculty of Science, Masaryk University (CZ 62760214). Fertilized ISA Brown chicken eggs were obtained from Integra farm (Zabcice, Czech Republic). Eggs were incubated in a humidified incubator at 37.8°C for 48 hours, and then all eggs were opened. Next, 2 ml of egg white was extracted for easier handling, and holes in the eggshell were secured by tape. No exclusion criteria were applied for experimental animals. At the selected time point, embryos were decapitated and fixed in 4% PFA overnight. Skulls and limbs were isolated from the embryos. Fixed tissues were processed through a dehydration ethanol gradient (30 to 50 to 80 to 90 to 95 to 100%) at RT (each for 1 hour) and then placed directly into the prepared BABB solution for 24 hours to achieve complete tissue clearing. Embryos were staged according to the method of Hamburger and Hamilton (*52*).

### Fish (*D. rerio*)

All work with zebrafish juveniles were approved by the Ministry of Agriculture, Czech Republic (45052/2020-MZE-18134 and 45055/2020-MZE-18134) and experimental exposure to dye by the Ministry of Education, Youth and Sports (MSMT-34667/2022-1) and State Veterinary Administration/Section for South Moravian Region (Animal Experiments Approval: CZ 62760214). Embryos were produced by natural spawning of AB zebrafish strain in breeding chambers after the preceding overnight separation of females and males. After spawning, embryos were collected and kept in an E3 medium with daily medium changes. No exclusion criteria were applied for experimental animals. From 1 DPF, 0.003% 1-phenyl-2-thiourea (P7629, Merck) was added to the medium to block the pigmentation process. Since 5 DPF, embryos were fed daily with dry food (ZebraFeed, Sparos). Once the embryos reached the desirable stage, they were transferred to a tank where the fluorochrome dyes were diluted in an E3 medium. Before imaging, embryos were anesthetized using buffered tricaine solution [tricaine-ethyl 3-aminobenzoate methane sulfonate salt (0.17 mg/ml) in 0.09 mM tris-HCl (pH 7.0)] (A5040, Merck). Whole-mount zebrafish were embedded in 1% TopVision Low Melting Point Agarose (R0801, Thermo Fisher Scientific).

### Microscopy and image analysis

Wide-field fluorescence overview images of specimens were taken on Axio Zoom V.16—Apotome (Zeiss). Detailed confocal images were obtained using the laser scanning confocal microscope (LSM 880, Zeiss), and overview visualizations of chameleon jaws were scanned using light sheet fluorescence microscopy (Lightsheet 7, Zeiss). The data were rendered and analyzed using Imaris (Bitplane) and ZEN (Zeiss) software.

### Statistical analysis

All the statistical analyses use the unpaired *t* test and were performed using the GraphPad Prism 8 (GraphPad Software, United States). Data are shown as the means ± SD. Statistically significant differences are presented in the figures as follows: *P < 0.05, **P < 0.01, ***P < 0.001, ****P < 0.0001, and “ns,” which is not significant.
