# Supplementary material for: Spatiotemporal monitoring of hard tissue development reveals unknown features of tooth and bone development
Source: Sci Adv. 2023 Aug 2;9(31):eadi0482. doi: 10.1126/sciadv.adi0482 (PMC10396306; doi:10.1126/sciadv.adi0482)
Supplement: Supplementary file 1 — Table S1 Legends for movies S1 to S3 [file sciadv.adi0482_sm.pdf]

Science Advances
AAAS

Supplementary Materials for

Spatiotemporal monitoring of hard tissue development reveals unknown features of tooth and bone development

Marcos Gonzalez Lopez *et al.*

Corresponding author: Jan Krivanek, jan.krivanek@med.muni.cz

Sci. Adv. **9**, eadi0482 (2023)
DOI: 10.1126/sciadv.adi0482

The PDF file includes:

Table S1
Legends for movies S1 to S3

Other Supplementary Material for this manuscript includes the following:

Movies S1 to S3

Table S1.

Volumes and ratios of administered dyes. In the mice, the administration was optimized according to the age of the animal. For aquatic species, the prepared dyes were directly diluted in the water or medium at the correspond ratios. (P – postnatal day).

	**Mouse** (*Mus musculus*)	**Chameleon** (*Chamaeleo calyptratus*)	**Chicken** (*Gallus gallus*)	**Frog** (*Xenopus laevis*)	**Fish** (*Danio rerio*)
**Alizarin Red S** (0.9 mg/ml)	50 µL (P0-P5)<br>100 µL (P6-P20)<br>200 µL (P21+)	200 µL	200 µL	1:30	1:500
**Calcein Green** (4.5 mg/ml)	50 µL (P0-P5)<br>100 µL (P6-P20)<br>200 µL (P21+)	200 µL	200 µL	1:10	1:500

Other Supplementary Material

Movie S1.

Root elongation of the first mouse mandibular molar. The mouse was alternatively injected with alizarin and calcein each 72 hours from P12 (postnatal day 12) to P30. The three-dimensional reconstruction obtained from confocal microscopy demonstrates how the elongation of molar roots is gradually slowing down.

Movie S2.

Crown formation of the first mouse mandibular molar. The mouse was injected by alizarin at P4 (postnatal day 4) and calcein at P6. Incremental growth lines in the crown area corresponding to the developmental days 4 and 6 can be analyzed.

Movie S3.

Development of the chameleon dentition. Juvenile chameleons were administered by alizarin at P14 (postnatal day 14), calcein at P28 and by the combination of both dyes at P42 (producing an orange color). The three-dimensional reconstruction allows to observe the dynamics of the fusion of the neighboring teeth and the interaction between tooth and underlying bone (fusing by ankylosis).
